# Conjugated Linoleic Acid Effects on Cancer, Obesity, and Atherosclerosis: A Review of Pre-Clinical and Human Trials with Current Perspectives

**DOI:** 10.3390/nu11020370

**Published:** 2019-02-11

**Authors:** Laura J. den Hartigh

**Affiliations:** Department of Medicine, Metabolism, Endocrinology, and Nutrition; UW Medicine Diabetes Institute, University of Washington, Seattle, WA 98109, USA; lauradh@u.washington.edu; Tel.: +1-206-543-8406

**Keywords:** conjugated linoleic acid (CLA), cancer, obesity, diabetes, atherosclerosis, gut microbiota

## Abstract

Obesity and its comorbidities, including type 2 diabetes and cardiovascular disease, are straining our healthcare system, necessitating the development of novel strategies for weight loss. Lifestyle modifications, such as exercise and caloric restriction, have proven effective against obesity in the short term, yet obesity persists because of the high predilection for weight regain. Therefore, alternative approaches to achieve long term sustainable weight loss are urgently needed. Conjugated linoleic acid (CLA), a fatty acid found naturally in ruminant animal food products, has been identified as a potential anti-obesogenic agent, with substantial efficacy in mice, and modest efficacy in obese human populations. Originally described as an anti-carcinogenic fatty acid, in addition to its anti-obesogenic effects, CLA has now been shown to possess anti-atherosclerotic properties. This review summarizes the pre-clinical and human studies conducted using CLA to date, which collectively suggest that CLA has efficacy against cancer, obesity, and atherosclerosis. In addition, the potential mechanisms for the many integrative physiological effects of CLA supplementation will be discussed in detail, including an introduction to the gut microbiota as a potential mediator of CLA effects on obesity and atherosclerosis.

## 1. Conjugated Linoleic Acids

Conjugated linoleic acids (CLAs) are polyunsaturated fatty acids that are found naturally in ruminant animal food products [1]. To date, there are at least 28 known isomers of CLA, defined by two conjugated double bonds in different geometric (i.e., *cis* or *trans*) and positional locations. Of these, 18:2*cis*-9, *trans*-11 (9,11 CLA, or rumenic acid), and 18:2*trans*-10, *cis*-12 (10,12 CLA) are the most naturally abundant, representing approximately 85% and 10% of all naturally occurring CLA isomers, respectively [2,3,4]. CLAs derive from the biohydrogenation of linoleic acid (18:2*cis*-9, *cis*-12) of bacteria that express linoleic acid isomerase, which encompass a large proportion of ruminant bacteria [5] (described in more detail in the Biohydrogenation of CLA section). Thus, food from ruminant sources, such as beef, lamb, butter, and dairy products, are natural sources of CLA. 

CLA can also be prepared synthetically from oils rich in linoleic acid, such as safflower, sunflower, corn, and soybean oils, taking advantage of the alkaline-catalyzed reaction that converts linoleic acid into CLA. Such a synthetic preparation renders a different proportion of the most common CLA isomers, yielding ~40%–45% 9,11 CLA, and ~40%–45% 10,12 CLA, with the remainder comprised of small amounts of other CLA isomers [6]. The synthetic derivation of CLA is often termed “mixed” CLA because of the approximately 1:1 ratio of 9,11 and 10,12 CLA.

As a result of the presence of one double bond in the *trans* configuration, CLAs are technically considered to be trans fatty acids. However, CLA is currently not classified as a trans-fat by the United States Food and Drug Administration (FDA). CLA was given a “Generally Regarded as Safe (GRAS)” designation by the FDA in 2008, exempting it from classification as a trans-fat on nutrition labels. In support of this, while studies in humans suggest that the consumption of industrially produced trans fats may be positively correlated with coronary heart disease risk factors [7,8,9,10,11], the consumption of ruminant-derived trans fats, such as CLA, does not [12,13], and may even be negatively associated with cardiovascular disease [7,14].

## 2. Biohydrogenation of CLA

Polyunsaturated fatty acids (PUFAs) such as linoleic acid (C18:2), which are abundant in typical livestock feed, grass, and hay, are toxic to many rumen bacteria [15,16,17]. Thus, the majority of dietary PUFAs undergo biohydrogenation in the rumen through a series of fatty acid intermediates, ultimately resulting in the generation of less toxic saturated fat (in this case stearic acid, C18:0). During this process of the biohydrogenation of PUFAs, such as linoleic acid by linoleic acid isomerase, intermediate by-products are also generated, such as 9,11 and 10,12 CLA [18]. The resulting lipid products of biohydrogenation, including saturated fatty acids and CLA isomers, thus become significant constituents of ruminant dairy and meat products.

The CLA content of ruminant food products is highly dependent on various factors, such as the type of feed [19,20], age and breed of the animal [21], environmental season [22], and the rumen pH [23,24,25]. Feed such as grass or corn contain higher levels of PUFA, and thus yield a higher CLA content in the animal [19,20,26,27,28,29]. The spring and summer months usually yield the highest CLA levels, reportedly nearly double the levels achieved during the winter [30,31,32]. Grain consumption decreases the rumen pH, thus reducing the abundance of the key rumen bacteria linoleic acid isomerase activity; conversely, grass feeding promotes a more favorable rumen environment for CLA-producing bacteria [28]. CLA consumption has many purported health benefits, which are described in later sections about anti-carcinogenicity, anti-obesogenicity, and anti-atherogenicity. As such, efforts to increase the CLA content of ruminant food products have been ongoing for some time. However, it was quickly observed that cows that were fed diets designed to increase their endogenous CLA content had a depressed milk fat production [18,33]. A study in which Holstein cows were fed diets supplemented with partially hydrogenated soybean oil, in which the intake of CLA was high, showed a significant correlation between the depression of milk fat and the ruminal content of trans_10_-18:1 [34], a fatty acid metabolite of which 10,12 CLA is a direct precursor. Subsequent studies concluded that the increased ruminant CLA production led to decreased de novo fatty acid synthesis in the mammary tissue of cows [35], the first indication that CLA inhibits lipogenesis, an effect now attributed to the 10,12 CLA isomer specifically [36]. CLA supplementation has also been shown to reduce milk fat content in humans by similarly inhibiting lipogenesis [37].

The fortification of dairy products, such as yogurt, milk, and cheese, with CLA (usually mixed CLA) is another method by which humans can increase their CLA consumption [38]; however, few studies have examined the impact of such CLA-fortified foods on human health. Fueled by the purported health benefits imparted by CLA as well as the booming nutraceutical industry, CLA has now been widely marketed as a nutritional supplement (discussed in more detail in the section on the “Anti-obesogenic effects of CLA” below).

## 3. CLA Effects on Cancer

### 3.1. Anti-Carcinogenicity of CLA in Murine Studies

One of the first beneficial effects attributed to CLA was its anti-carcinogenicity, discovered by Michael Pariza. Synthetically prepared CLA isomers were applied topically to mice prior to the initiation of epidermal carcinomas. Mice that received topical CLAs developed only half the number of papillomas, and exhibited a lower incidence of tumor formation relative to the control mice [39]. Subsequent studies have shown that other murine carcinoma models show an improvement with CLA supplementation, including mammary [40,41], colon [42,43,44,45], stomach [42], prostate [46], and hepatic carcinomas [47,48,49]. The additional potential mechanisms for the anti-carcinogenicity of CLAs are that it induces apoptosis in tumor cells (indicated by a reduced Bcl-2 expression and increased terminal deoxynucleotidyl transferase (TdT) dUTP nick-end labeling (TUNEL assay) and Annexin-V staining) [50,51,52], it exhibits anti-angiogenic, antioxidant (reduced reactive oxygen species and peroxide generation) [42,52], and altered arachidonic acid metabolism [53,54,55], and it has been shown to promote anti-proliferation [41,56].

Contrary to the initial studies suggesting that CLA is anti-carcinogenic, some studies have shown that CLA has no effect on tumor inhibition, and a few have even shown that CLA promotes tumor progression. Wong et al. did not find an effect of mixed CLA (up to 0.9%) supplementation on established aggressive mammary tumors in mice [57]. Similarly, a higher dose of mixed CLA (3%) did not reduce the tumor burden in *APC^Min/+^* mice [58], a genetic model that spontaneously develops intestinal tumors [59]. A pilot study in mice suggested that mixed CLA supplementation increased tumor progression in PyMT transgenic mice, a mouse model of invasive breast cancer [60], and 10,12 CLA specifically has been shown to increase tumorigenesis in transgenic mice overexpressing ErbB2 in mammary epithelium, another murine model of mammary carcinoma [61,62]. While a handful of studies suggest that CLA has no effect on, or even worsens tumor progression, the majority of studies show beneficial effects of CLA supplementation. It is likely that the timing of the CLA supplementation is critical, and that CLA is most effective as an anti-carcinogen when administered during early tumorigenesis, and is less effective in models of established tumors.

### 3.2. CLA Studies on Cancer in Humans

There is some evidence to suggest that CLA consumption reduces the incidence and progression of some types of cancer in humans. There is a significant negative correlation between milk intake and risk of breast or colon cancer [63], an effect that is coincident with elevated serum CLA levels in a particular group of Finnish women [64]. Similarly, another study showed that subjects consuming four or more servings of dairy per day showed a reduced risk of colorectal cancer [65]. Furthermore, a study was conducted in women with Stage I–III breast cancer, in which the subjects (*n* = 23, no placebo group) were given 7.5 g/day mixed CLA for at least 10 days prior to their tumor removal surgery [66]. Spot 14 (S14), a regulator of fatty acid synthesis that has been shown to augment breast cancer proliferation [67], was decreased following CLA supplementation [66]. Similarly, the Ki-67 scores declined with CLA treatment, indicative of a reduction in tumor proliferation [66]. The results of these studies suggest that CLA could be a potential therapeutic against breast and/or colon cancer.

Contrary to the handful of studies touting the anti-carcinogenicity of CLA, additional studies performed by different groups on different cohorts of French and American women failed to show any correlation between CLA and incidence of breast cancer [68,69,70,71]. Thus, with mixed results and a low number of studies, there is insufficient evidence to determine whether CLA ingestion has a significant effect on cancer.

### 3.3. Effects of CLA on Body Weight and Composition

#### 3.3.1. CLA Supplements

Approximately 15% of adults in the United States report the use of non-prescription dietary supplements to promote weight loss [72], contributing to the billion-dollar nutraceutical industry that is not heavily regulated by the FDA. As such, the consumers of dietary supplements are at risk of side effects, variable efficacy, and adverse interactions with medications that may not be rigorously tested for nor acknowledged by nutraceutical manufacturers. With purported health benefits such as the promotion of weight loss, CLA has been marketed as a supplement to promote weight loss. CLA supplements are mass produced synthetically from safflower oil and are encapsulated [73], and are readily available for purchase from many sources. Given that CLA supplements contain an equal proportion of 9,11 CLA and 10,12 CLA, the effects of CLA supplementation on body energetics can be dramatically different than the effects of CLA procured from the natural diet.

#### 3.3.2. CLA Effects on Obesity in Animal Models

The dramatic effect CLA has on adiposity has been studied for the past 20 years. Initial studies in male and female ICR mice showed that a mixed CLA (0.5% *w*/*w*)-supplemented chow diet promoted fat loss by 60% over 30 days [74], attributed to increased lipolysis and fat oxidation [74,75]. Similarly, an early study in Sprague-Dawley rats noted that the triglyceride and non-esterified fatty acid levels were reduced in white adipose tissue in a dose-dependent manner, following three weeks of mixed CLA feeding [76]. Importantly, both of these initial observations were made before there was an observable change in body weight. It was later determined that the active CLA isomer responsible for reducing adiposity was 10,12 CLA [75]. The initial adiposity-lowering effect of mixed CLA has been confirmed many times in various mouse models [77,78,79,80], rats [81], hamsters [82,83], and pigs [84,85,86]. However, it is likely that CLA supplementation impacts distinct species, such as mice and rats, differently. Lean Zucker rats fed a 0.5% mixed CLA-containing diet for five weeks showed a reduced adiposity, while obese Zucker rats showed an enhanced adiposity [87]. The reason for this species difference is not clear, but could relate to differences in study design.

Much work has been devoted to understanding the mechanism(s) by which 10,12 CLA promotes fat loss in rodent models. Early studies suggested that 10,12 CLA promotes the oxidation of fat stores, assessed by measuring the gene expression of peroxisome proliferation activated receptor alpha (*Ppara*), and its downstream targets acyl coenzyme A oxidase (*Acox1*) and carnitine palmitoyl transferase-1 (*Cpt1*) [88,89,90]. Recently, work in my lab has expanded on this observation, by showing that 10,12 CLA robustly increases the conversion of radiolabeled fatty acids, such as palmitate and oleate, into carbon dioxide in cultured adipocytes [91], suggestive of enhanced fatty acid oxidation. Moreover, we have also shown in vivo that acylcarnitines, major byproducts of fatty acid oxidation, are robustly increased in mice receiving 10,12 CLA supplementation [80]. More recent evidence points towards the browning of white adipose tissue as a mechanism of fat mobilization, to promote weight and fat loss [80,92,93,94]. The first study, by Wendel et al., to suggest that CLA (1.5% mixed isomer) promoted UCP1 expression in white adipose tissue was performed in *ob*/*ob* mice, a mouse model of spontaneous obesity due to the deletion of the leptin gene [92]. Importantly, this study only reported white adipose tissue browning in epididymal (visceral) white adipose tissue, and this effect of CLA was noted during the obesogenic period. The next group to examine the browning effect of CLA in detail used Sv129 mice given low-doses of CLA isomers. This group, led by Michael McIntosh, showed that several low doses of 10,12 CLA (0.03%, 0.1%, and 0.3%) promoted the increased uncoupling protein 1 (UCP1) expression from both inguinal (subcutaneous) and epididymal (visceral) white adipose tissue in lean mice [94]. This group went on to show that 10,12 CLA at the 0.1% dose could similarly promote white adipose tissue browning during low-fat diet feeding [93]. In the study by Shen et al., 10,12 CLA was given at a dose that was approximately six-fold lower than the study by Wendel et al., yet promoted the browning of both epididymal (visceral) and inguinal (subcutaneous) white adipose tissue with minimal detrimental effects on liver weight [93,94]. More recently, our group has shown that in a mouse model with phenotypes resembling human metabolic syndrome (*Ldlr*^−/−^ mice fed a diet high in sucrose and saturated fat), 10,12 CLA (1%) promotes the substantial browning of white adipose tissue in obese mice, with coincidently increased energy expenditure [80]. The mice were also able to better maintain a core body temperature when challenged with exposure to the cold, suggesting that the browning of white adipose tissue could be involved with thermogenesis. Further studies are thus warranted to determine if the browning effects of 10,12 CLA are causal for its weight loss effects.

Several groups have shown that CLA supplementation reduces the circulating levels of adiponectin, a hormone secreted from adipocytes that has anti-inflammatory [95], anti-atherogenic (reviewed in [96]), and anti-diabetic properties [97,98,99]. Recently, it has become recognized that while CLA supplementation reduces fat stores, it also dramatically decreases the circulating adiponectin levels in mice [80,100], which could explain why CLA-mediated weight loss is associated with impaired glucose metabolism. The reason for this loss of adiponectin is unclear, but a recent study by our group suggests that by at least partially restoring the adiponectin levels, co-treatment with the thiazolidinedione rosiglitazone, a peroxisome proliferator activated receptor gamma (PPARγ) agonist that directly upregulates adiponectin [101], also improves glucose metabolism, while still allowing for CLA-mediated weight loss [102]. While some studies suggest that CLA is a PPARγ agonist [103,104,105,106,107], the majority of studies to date have shown that CLA decreases PPARγ mRNA expression, activation, and downstream targets [100,108,109,110,111,112]. Thus, we are beginning to understand that CLA promotes a form of weight loss that is not especially metabolically healthy, as evidenced by the reduced PPARγ activity and adiponectin levels. Mechanistically, this can be alleviated by co-therapies that mitigate the negative effects on glucose metabolism.

#### 3.3.3. CLA Weight Loss Effects in Humans

CLA studies in humans are difficult to interpret because of small sample sizes, variable doses and isomers of CLA, a wide range of supplementation durations, and study population characteristics. All of the human trials conducted with CLA to date that used obesity metrics as an endpoint are listed in Table 1, and are to be highlighted in this section. The first trials to examine the weight loss properties of supplemental CLA in humans were conducted by a Norwegian group led by Ola Gudmundsen [113,114]. These randomized, double blind, placebo-controlled studies recruited healthy men and women with a body mass index (BMI) between 25 and 35 kg/m^2^. In one study, participants were instructed to consume placebo (9 g olive oil, *n* = 10) or mixed CLA supplements daily for 12 weeks at one of the following four doses: 1.7, 3.4, 5.1, or 6.8 g CLA/day (*n* = 12, 8, 11, and 11 participants, respectively) [113]. The subjects that received CLA had a significantly higher reduction in body fat mass in a dose-dependent manner, without noticeable differences in BMI or lean mass [113,114], suggesting that adiposity and not body weight per se was influenced by CLA. The same group extended these initial studies, using a similar design, for one year of CLA supplementation (*n* = 150), and found that subjects again lost fat mass, and this time, they also significantly lost body weight [115]. Another study led by Dale Schoeller yielded similar results. This study examined the ability of CLA to curb holiday-associated weight gain. In this randomized, double-blind, placebo-controlled study, 40 healthy, overweight men were given either placebo or 3.2 g/day mixed CLA for six months, from August through March^118^. Compared with the placebo, the CLA group lost body fat, with no evidence of adverse liver function, inflammation, or insulin resistance [116]. Similarly, a study of overweight men and women with early symptoms of metabolic syndrome (MetS) (subjects had to meet two criteria for MetS, such as elevated blood pressure, fasting glucose, or triglycerides, or low high-density lipoproteins (HDL) cholesterol) showed that 3 g mixed CLA per day decreased adiposity without any noted deleterious effects on glucose or liver metabolism [117]. These studies and many others (listed in Table 1) have reported a beneficial effect of CLA supplementation on parameters related to body weight and/or adiposity, with no evidence of negative metabolic consequences.

In addition to adults, CLA appears to have efficacy in overweight children. In a randomized, double-blind, placebo-controlled study, 53 healthy children (aged 6–10 years) who were overweight or obese received 3 g/day mixed CLA or placebo for six months [126]. The children that received CLA had lower body fat, but did not have improved plasma lipids or glucose levels. Notably, the fat lost from these children was largely from the peripheral compartment, with visceral fat being maintained.

While CLA has been shown in many studies to have clinical benefit related to body weight and adiposity, many subsequent studies have failed to show any effects of mixed CLA supplementation on body metrics [127,136,142,143]. One potential reason that fat loss was achieved in the Norwegian studies relates to the study design. Participants were instructed to consume their daily dose of CLA in three increments, one increment with each meal throughout the day. It is possible that a continuous dosing strategy is important for human efficacy. Another possibility is that the doses of the active isomer of CLA, 10,12 CLA, were higher in the studies by Gudmundsen et al. Significant differences in the basal metabolic rate between mice and humans predicted that CLA-mediated reductions in body fat would be seven times higher in mice than humans [144], which could explain the lack of efficacy often reported in human CLA studies. The studies by Gudmundsen et al. used higher doses of 10,12 CLA than other studies at the time [144], which could explain their beneficial findings on adiposity.

A Swedish group, led by Bengt Vessby, conducted several human trials, suggesting that mixed CLA and individual CLA isomer supplementation promote deleterious side effects in addition to body weight and fat loss. This group initially showed that 4.2 g/day mixed CLA effectively reduced the sagittal abdominal diameter in obese men after four weeks [133]. However, further studies by this group also showed, for the first time, that the 10,12 CLA isomer could promote insulin resistance. Abdominally obese men (*n* = 60 total) were given 3.4 g mixed CLA/day, 3.4 g/day purified 10,12 CLA, or placebo for 12 weeks [134,145]. The group given 10,12 CLA lost significant body weight and body fat, but also exhibited elevated plasma insulin and glucose, reduced insulin sensitivity, elevated C-reactive protein (CRP) levels, and increased urinary prostaglandin 8-iso-PGF (2alpha), indicative of oxidative stress [134,145,146]. These studies represented the first evidence that 10,12 CLA-mediated weight loss might occur at the expense of metabolic health; however, it is important to note that the Swedish studies did not measure insulin resistance directly.

### 3.4. Effects of CLA on Glucose Metabolism and Diabetes

With several reports of the negative effects of CLA supplementation on indices of glucose metabolism, subsequent studies began to examine the effects of CLA supplementation in study populations with impaired glucose metabolism. Subjects with type 2 diabetes mellitus who were given 8 g/day mixed CLA for eight weeks showed an inverse relationship between the plasma CLA levels and body weight, an effect attributed to the 10,12 isomer, which suggests that CLA retains efficacy at lowering body weight in patients with insulin resistance [147]. In another study, obese children without clinically diagnosed diabetes were given either a placebo, mixed CLA (3 g/day), or metformin (1 g/day) for 16 weeks (*n* = 14–18 children/group) [141]. Importantly, the supplementation or medication was spaced out throughout the day with three doses per day. No treatment effects were observed regarding body weight, BMI, or waist circumference. However, the subjects given CLA exhibited improved indices of glucose tolerance, such as reduced fasting insulin and homeostatic model assessment of insulin resistance (HOMA-IR), suggesting that CLA may improve obesity-associated insulin resistance in children [141]. These studies suggest that CLA supplementation in diabetic-susceptible populations may improve glucose metabolism, notably in children.

Despite a handful of studies suggesting beneficial glucose metabolic effects in humans, other studies also show no clinical benefit. A six-month randomized, double-blind, placebo-controlled study in overweight and obese men and women showed that six months of mixed CLA supplementation (3.4 g/day) did not significantly alter the glucose metabolism or insulin sensitivity, determined by hyperinsulinemic euglycemic glucose clamps with a homeostasis model assessment (HOMA) [148]. Furthermore, a study of 55 obese postmenopausal women with type 2 diabetes participated in a randomized, double-blind, cross-over study of mixed CLA or safflower oil supplementation for 16 weeks, with a four-week washout period between supplements [123]. CLA was administered at a dose of 8 g/day, and study participants were instructed to consume 2 g with each meal and one before bed, thus spacing out their CLA consumption. The CLA effectively lowered the BMI and total adipose mass, but was not effective at improving the indices of glucose metabolism, such as fasting glucose, insulin, adiponectin, and HOMA-IR, all of which were improved with safflower oil. The results of this study suggest that while CLA promotes weight and fat loss in humans, it does not confer significant beneficial effects on glucose metabolism.

Moving on to animal models, initial studies in diabetic Zucker fatty rats suggested that two weeks of mixed CLA supplementation (1.5% *w*/*w*) promoted a beneficial effect on glucose metabolism by normalizing the glucose tolerance and reducing the fed and fasting glucose levels [149]. Subsequent studies have supported this effect in the rat, and attributed it to a beneficial effect of the 10,12 CLA isomer [150,151]. However, a wealth of more recent studies suggest that CLA, in particular the 10,12 CLA isomer, promotes insulin resistance. Female C57Bl/6J mice given a large dose of mixed CLA (2.75%) not only exhibited marked reductions in adiposity, but also severe derangements in glucose and insulin tolerance [152]. Similarly, male AKR/J or C57Bl/6J mice had worsened plasma insulin levels after 1% or 0.4% (*w*/*w*) mixed CLA supplementation, respectively [153,154]. Whether such discrepancies are due to different species (mice vs. rats) or different CLA doses and preparations, remains to be determined.

There is some evidence that co-therapies including CLA and insulin sensitizing agents could be a plausible strategy against obesity. The Belury group were the first to show that co-treatment with mixed CLA and the thiazolidinedione rosiglitazone, a PPARγ agonist that promotes insulin sensitivity and is used clinically to treat type 2 diabetes, improves insulin sensitivity in mice that are undergoing weight gain [155]. We later showed that co-treatment with rosiglitazone and 10,12 CLA (1%) not only promotes weight loss in obese low-density lipoprotein-deficient mice (*Ldlr*^−/−^ mice), but improves obesity-associated insulin resistance while promoting a favorable redistribution of the adipose stores toward the subcutaneous sites [102]. Taken together, the available evidence suggests that while CLA can modestly reduce the total body mass and regional body fatness, these changes do not provide the metabolic benefit of improved glucose metabolism that would be expected with weight and fat loss. However, to date most human trials have not reported a worsening in the indices of glucose metabolism, with the exception of the Vessby group in Sweden.

### 3.5. Effects of CLA on Atherosclerosis

#### 3.5.1. Pathology of Atherosclerosis

Cardiovascular disease is a major cause of death in most developed countries, and most cardiovascular events, such as heart attack and stroke, are secondary to atherosclerosis. Atherosclerotic plaques are characterized by the accumulation of lipids and inflammatory cells in the arterial wall, which can become progressively less stable, leading to rupture and subsequent ischemic events. The detailed mechanisms of atherosclerotic development are reviewed in the literature [156]. Effective treatment strategies for atherosclerosis include medications such as statins, ezetimibe, or niacin to lower blood cholesterol; fibrates to lower blood triglycerides; and aspirin to reduce inflammation; lifestyle treatment includes changes such as an improved diet and increased exercise; and surgical options include angioplasty, endarterectomy, and vessel bypass grafting. Recently, alternative approaches for the treatment of atherosclerosis have become more widespread, including certain food-derived products and supplements, such as fish oil, folic acid, garlic, omega-3 fatty acids, psyllium, coenzyme Q10, black or green tea, and oat bran. In this section, the effects of CLA on atherosclerosis development and progression will be described, making a case for adding it to the list of potential anti-atherosclerotic supplements.

#### 3.5.2. Initial Studies in Rabbits and Hamsters

The first studies to test the atherogenicity of CLA supplements were performed in rabbits, a species that readily develop human-like atherosclerosis when fed a cholesterol-containing diet (reviewed in the literature [157]). Mixed CLA at doses ranging from 0.05%–1% consistently show reduced aortic lipid deposition [158], regression of established lesions [159,160], and improved plasma lipoproteins during regression [160]. Because of the higher cost required to perform these types of studies in rabbits, and the ease of genetic modification of mice, subsequent studies primarily utilized rodent models to test the effects of CLA supplementation on atherosclerosis.

The Syrian Golden hamster is another useful model for studies of atherosclerosis because of its similar lipoprotein profile to humans when fed a diet high in fat and cholesterol [161]. Initial studies suggested that mixed CLA supplementation in hamsters fed either a chow or high-fat diet with added cholesterol did not alter atherosclerosis development when compared to linoleic acid-supplemented controls [162,163], despite reported improvements in the plasma lipids [164,165,166]. Thus, while CLA supplementation may improve some parameters associated with atherosclerosis, it does not appear to contribute to atherosclerosis regression in the hamster. Importantly, while the lipoprotein profile of the hamster more closely resembles that of humans, the atherosclerotic lesions are less severe than in other animal models, similar to early fatty streak-style lesions, representative of the early stages of atherosclerosis.

### 3.6. Effects CLA on Atherosclerosis in Mice

While mice inherently have a much different lipoprotein profile that of humans, where the majority of the cholesterol is carried on HDL rather than both low-density lipoproteins (LDL) and HDL, mice with particular genetic and dietary manipulations can develop atherosclerotic plaques that closely resemble human atherosclerosis (reviewed in the literature [167]). Two of the most common mouse strains used for the study of atherosclerosis are apoE-deficient mice (*apoE*^−/−^) and *Ldlr*^−/−^ mice. Both models develop atherosclerosis as a result of the accumulation of cholesterol-containing lipoproteins in the plasma due to defective lipoprotein clearance. *ApoE*^−/−^ mice develop spontaneous atherosclerotic lesions that closely resemble those of humans [168], but because of the extreme changes in the lipoprotein levels that are exacerbated by high-fat- and/or cholesterol-containing diets, this model is somewhat less relevant to human disease. Moreover, the apoE protein not only functions in lipoprotein clearance, but also plays important roles in modulating inflammatory responses [169]. Thus, the *apoE*^−/−^ model promotes atherosclerosis by both lipid- and inflammation-mediated pathways. By contrast, the *Ldlr*^−/−^ model mimics the elevated cholesterol levels observed in human familial hypercholesterolemia when mice are fed a high-fat, cholesterol-containing diet. Unlike the *apoE*^−/−^ mouse, which carries the majority of the excess cholesterol in very low-density lipoproteins (VLDL) and remnant lipoproteins, the *Ldlr*^−/−^ mouse carries the excess cholesterol in the low-density lipoprotein (LDL) fraction, which is more similar to humans. Also, similarly to humans, the atherosclerotic lesions in the *Ldlr*^−/−^ mice progress from early fatty streaks to complex lesions over time. Double *apoE*^−/−^*Ldlr*^−/−^ mice develop comparable levels of hypercholesterolemia and lipoprotein profiles as *apoE*^−/−^ mice [170], but with more extensive atherosclerosis that more closely resemble human plaques [171]. With the key differences in the lipoprotein profiles and plaque structure, studies comparing the outcomes from these mouse models should be interpreted with caution. To date, most studies examining the effects of CLA supplementation on atherosclerosis have been performed in *apoE*^−/−^ mice, somewhat limiting the translational knowledge gleaned by these mouse studies.

While there has yet to be consensus on the effect of CLA on atherosclerosis, the majority of CLA supplementation studies in *apoE*^−/−^ mice show a suppression or regression of atherosclerosis. Several studies using mixed CLA supplementation strategies have found reduced atherosclerotic lesion sizes in the aorta [172,173], coupled with decreased macrophage accumulation and decreased expression of pro-inflammatory genes [172]. *ApoE*^−/−^*Ldlr*^−/−^ double knockout mice fed eggs supplemented with 0.1% mixed CLA also exhibited protection from atherosclerosis with a coincident decreased plaque macrophage content [174]. These effects of CLA supplementation on atherosclerosis appear to be mediated primarily by the 10,12 CLA isomer. *Ldlr*^−/−^ and *ApoE*^−/−^ mice fed a low-fat semi-purified diet containing 0.12% cholesterol, with 0.5% of either 10,12 CLA or mixed CLA (containing approximately 40% 10,12 CLA) for 11 weeks, developed less en face and aortic root atherosclerosis than the mice fed 9,11 CLA, suggesting that 10,12 CLA is the isomer responsible for atheroprotection [175]. Such an inhibition of atherosclerosis occurred despite a worsened hepatic metabolic profile, including increased liver weight and triglycerides [175], an effect seen previously in several studies [80,174]. Similarly, our study using obese *Ldlr*^−/−^ mice that had been fed a 0.15% cholesterol-containing high-fat high sucrose diet showed significant improvements in aortic and sinus atherosclerosis when given 10,12 CLA (1% *w*/*w*), an effect that was independent from weight loss, as a weight-matched control group undergoing caloric restriction did not exhibit such improvements in atherosclerosis [176]. In this work, we also found that the perivascular adipose tissue surrounding the thoracic aorta was enriched with macrophages that could be classified as “alternatively-activated”, “resident”, or “M2”, exhibiting high expression levels of Arginase-1 (*Arg1*) and an early growth response-2 (*Egr2*) [176]. Subsequent in vitro experiments showed that 10,12 CLA polarized bone marrow-derived macrophages towards this alternative phenotype [176], which has been shown to confer protection against atherosclerosis [177,178]. We had previously reported that white adipose tissue becomes enriched with alternatively-activated macrophages in mice undergoing weight loss by 10,12 CLA supplementation [80]. This raises the intriguing possibility that 10,12 CLA’s profound effects on adipose tissue, in this case to enrich the adjacent perivascular adipose tissue with resident macrophages, could provide protection from atherosclerosis through the modulation of the aortic microenvironment. Collectively, these studies and others suggest that CLA, and likely 10,12 CLA specifically, protect against atherosclerosis, an effect that may be independent from the weight-loss effects of CLA.

There are a few studies suggesting that CLA supplementation has little effect on atherosclerosis. In one such study, *apoE*^−/−^ mice fed mixed CLA for 12 weeks showed no improvement in either en face or aortic sinus atherosclerosis [179]. Similarly, neither the atherosclerosis progression nor regression were altered in the *apoE*^−/−^*Ldlr*^−/−^ double knockout mice given 0.5% 10,12 CLA [180]. Moreover, one study suggests that 10,12 CLA actually worsens aortic sinus atherosclerosis levels in *apoE*^−/−^ mice [181]. Thus, while the majority of studies published to date suggest an anti-atherosclerotic effect of CLA, true consensus on this effect has not yet been reached.

### 3.7. Effects of CLA on Lipids and Reverse Cholesterol Transport/Cholesterol Efflux

Cholesterol metabolism is central to atherosclerosis development. Reverse cholesterol transport is the process by which cholesterol is transported from the peripheral tissues to the liver for disposal and removal from the body [182]. The efflux of cholesterol from peripheral cells, such as macrophages or adipocytes, to high-density lipoproteins (HDL), is the first step in reverse cholesterol transport, and a critical process for the prevention of atherosclerosis. Thus, treatment strategies to increase reverse cholesterol transport and/or cholesterol efflux to HDL could lead to atherosclerosis regression.

There is some evidence that CLA promotes notable changes to HDL metabolism in vivo. CLA has been shown to reduce plasma cholesterol levels [174,176,183,184] and to increase HDL levels in mice [183,185,186], suggesting that CLA could impact cholesterol efflux. Some studies in vitro support this notion. Human peripheral blood mononuclear cell-derived macrophages and RAW264.7 macrophages respond to mixed CLA and 10,12 CLA by increasing the expression of ATP binding cassette transporter A1 (ABCA1), a major receptor for apolipoprotein A1 (ApoA1)-mediated cholesterol efflux, an effect associated with decreased macrophage cholesterol content and increased cholesterol efflux [187,188]. However, other in vitro studies suggest that CLA isomers may not directly influence the cholesterol efflux from macrophages. In cultured THP-1 macrophages, both 9,11 and 10,12 CLA increase the cluster of differentiation 36 (CD36) mRNA and expression, and similarly increased intracellular cholesterol content [189]. Additional studies suggest that neither CLA isomer influences the ATP binding cassette transporter A1 (ABCA1) expression [190] or cholesterol efflux to apoA1 [189]; thus, the CLA effects on macrophage cholesterol homeostasis in vitro have not yielded clear results. Further work is needed to determine whether the CLA isomers impact the cholesterol metabolism, and whether such effects influence atherosclerosis.

### 3.8. Effects of CLA on Monocytes and Macrophages

Monocytes and macrophages play pivotal roles in the development of atherosclerotic lesions. The response to injury hypothesis of atherosclerosis, initially described by Russel Ross [191], suggests that inflamed endothelial cells lining atherosclerosis-prone arteries recruit circulating monocytes, which are retained in the vessel wall and can become lipid-laden foam cells, an initial step in atherogenesis. Decades of subsequent work suggests that the macrophage phenotype is important for atherosclerosis progression, severity, and regression, and that dietary factors such as CLA can influence the macrophage phenotype.

CLA has several reported effects on endothelial cells, monocytes, and macrophages cultured in vitro; 9,11 and 10,12 CLA both have been shown to blunt the expression of the key monocyte adhesion molecules present on endothelial cells; vascular cell adhesion molecule-1 (VCAM-1) and intercellular adhesion molecule-1 (ICAM-1), with the additional suppression of the complimentary integrins very-late antigen 4 (VLA-4, comprised of CD49d/CD29) and macrophage-1 antigen (Mac-1, or CD11b), present on monocytes, resulting in a diminished adhesion to the endothelial cells [192]. In addition, mixed CLA blunts the cluster of differentiation 18 (CD18) surface expression and CXC chemokine receptor 4 (CXCR4) expression on human peripheral blood mononuclear cells, resulting in suppressed monocyte adhesion in a mouse model of atherosclerosis [193]. Furthermore, macrophages exhibit a reduced inflammatory profile in response to both 9,11 CLA and 10,12 CLA [176,194,195]. Collectively, evidence suggests that CLA isomers may have anti-inflammatory effects on monocytes and macrophages in vitro that could influence atherogenicity in vivo.

Traditionally, macrophages have been characterized phenotypically as classically (M1), alternatively (M2), or recently as metabolically (MMe) activated [196,197]. This classification strategy is undoubtedly oversimplified, but will suffice for the purposes of this review. It has been shown that the phenotypic switch from M2 to M1 occurs during atherosclerotic plaque progression [198], and that the M1 macrophage content of the atherosclerotic plaques are associated clinically with the incidence of ischemic events [199]. Conversely, M2 macrophages are associated with atherosclerosis regression and plaque stability [177,199]. In line with its potential atheroprotection, CLA has been shown to promote an M2 phenotype in cultured macrophages [176,200] and in atherosclerotic lesions in mice [201]. Moreover, 10,12 CLA specifically has been recently shown to promote the enrichment of adipose tissue with M2 macrophage markers arginase-1 (ARG-1) and early growth response protein-2 (EGR-2) in mice [80], including the perivascular adipose tissue adjacent to the aortic lesions [176], suggesting that signaling within the microenvironment of the atherosclerotic plaques could be important in atheroprotection. Taken together, mixed CLA and individual CLA isomers may impart atheroprotection by modulating the macrophage phenotype.

### 3.9. CLA effects on Cardiovascular Disease in Humans

While the available evidence suggests that CLA is protective against atherosclerosis in rabbits and mice, studies pertaining to cardiovascular disease in humans are a bit sparser and less informative (see Table 2 for a list of human studies). Several small-scale clinical trials have yielded underwhelming results for CLA efficacy against human disease. In the first, a double-blind, randomized, placebo-controlled trial investigated the effect of six months of 9,11 CLA supplementation (2.5 g/day 9,11 CLA + 0.6 g/day 10,12 CLA, *n* = 401 participants) on the clinical parameters related to atherosclerosis, including aortic pulse wave velocity, blood pressure, plasma lipids, and CRP levels [202]. There were no effects on any of the parameters measured using this CLA supplementation protocol; however, numerous studies in animals suggest that the 10,12 CLA isomer is primarily responsible for atheroprotection, which could explain the lack of improvement observed in this study. In addition, the participants recruited for this study were on average healthy, exhibiting few baseline risk factors for cardiovascular disease—mild hypercholesterolemia with normal triglyceride levels, blood pressure, and CRP levels. Perhaps conducting this study in a population with a higher cardiovascular disease risk would have yielded more clinical benefit.

The next study administered mixed CLA to overweight men (BMI 25–25 kg/m^2^, 4.5 g/day, *n* = 85 subjects) for four weeks [203]. There were no impairments in the endothelial function, measured by the peripheral arterial tonometry (PAT) index determination, nor changes in cardiovascular risk factors such as the platelet-activating factor acetylhydrolase (PAF-AH) activity, C-reactive protein, paraoxonase, LDL or HDL cholesterol, or triglycerides. In a similar study, overweight subjects (n = 74) were randomly assigned to either a placebo (sunflower oil) or mixed CLA (3 g/day) for 12 weeks [207]. The measures indicative of atherosclerosis, including asymmetrical dimethylarginine (ADMA) and high sensitivity C-reactive protein (hs-CRP), did not differ between groups, suggesting that mixed CLA did not improve the markers associated with atherosclerosis in this particular population^206^. Collectively, these two studies suggest that short-term CLA supplementation in moderately overweight men does not exacerbate cardiovascular risk factors.

In another small trial, human subjects with diagnosed coronary artery disease were given either 3 g/day mixed CLA or a placebo (*n* = 30 each), for two months. In this study, the first and only study conducted in human subjects with known cardiovascular disease, CLA had no effect on the plasma triglycerides, LDL cholesterol, or HDL cholesterol [213]. Thus, a limited number of human trials have failed to show a protective effect of CLA against cardiovascular disease risk factors. Notably, none of the trials conducted to date focused exclusively on the 10,12 isomer of CLA.

## 4. The Gut Microbiome: A New Target for CLA Production and/or Action

Ruminant animal food sources, such as beef and dairy products, are the primary sources of CLA found naturally in our diet. As discussed in the section on biohydrogenation, this is due to the abundance of CLA-producing bacteria found naturally in the rumens of animals fed a diet high in linoleic acid, including species of *Butyrivibrio*, *Lachnospiraceae*, *Bulleidia*, and *Coriobacteriaceae* [214,215,216,217]. Enrichment with these species in the rumens of dairy cows positively and strongly correlates with the 10,12 CLA milk content [214].

To date, a number of non-ruminant bacterial species are known to be capable of synthesizing CLA, including strains that are naturally present in the human gastrointestinal tract, such as *Lactobacillus*, *Bifidobacterium*, *Roseburia*, *Propionibacterium*, *Pediococcus*, *Enterococcus*, and *Lactococcus* [218,219,220,221,222,223,224,225,226,227]. These bacteria express the enzyme linoleic acid isomerase in a similar manner as the ruminant bacteria, to enable the conversion of linoleic acid to 9,11 CLA. In contrast, only a handful of studies have reported on gastrointestinal tract bacteria that can produce 10,12 CLA, including species of *Lactobacillus* and *Bifidobacteria* [224,225,228]. Moreover, CLA production has recently been confirmed in bacterial species isolated from the human gut [229,230,231,232], suggesting that strategies to increase the endogenous CLA production from the human microbiome would be feasible.

Several studies have shown that supplementing diet with 10,12 CLA specifically can alter the gut microbiota composition and associated gut metabolites. The first study to examine microbiome changes in response to oral 10,12 CLA supplementation (0.5% *w*/*w* in lean chow-fed mice for eight weeks), showed that despite no differences in body weight, adiposity was lower in the 10,12 CLA-treated group, and the concentrations of bacterial-derived short chain fatty acids (SCFA) were elevated in the cecae [233]. This elevation in the microbial metabolites was associated with notable but minor changes to the gut microbial composition, including reduced levels of *Firmicutes* and increased *Bacteroidetes* [233]. This suggests that low levels of 10,12 CLA supplementation can promote small changes in the gut microbial composition, which could impact the host metabolism. A subsequent study conducted by us showed that a higher dose of 10,12 CLA (1% *w*/*w*) in *Ldlr*^−/−^ mice fed a diet high in saturated fat and sucrose, an obese mouse model with features of human metabolic syndrome, and a model in which weight and fat loss were more prominent, also resulted in clear gut microbial changes [234]. In this study, higher levels of weight loss elicited by 10,12 CLA yielded significant elevations of the SCFAs butyrate in the feces and acetate in plasma, and were associated with changes in the composition of the gut microbiota, including an enrichment in species of *Butyrivibrio*, *Roseburia*, and *Lactobacillus*, all known producers of butyrate [234]. Future studies will determine whether such microbial changes are required for 10,12 CLA-mediated weight and fat loss, which could inform future treatment strategies for weight loss.

In addition to the effects on the distal gut microbiota, dietary CLA also has potential impact at the level of the upper gastrointestinal tract. It has recently been shown that CLA can become nitrated when it comes in contact with nitrates in saliva and acidic stomach contents [235,236], and nitrated CLA is detectable in the plasma and urine of healthy humans [235,237]. Nitrated-CLA has been shown to reduce the NFκB-dependent inflammatory gene expression, with subsequent decreases in inflammation in macrophages in vitro [238], an effect recapitulated by the decreased pro-inflammatory cytokines and decreased leukocyte recruitment in a mouse model of peritonitis [238]. Whether nitrated-CLA impacts the distal gut microbiota is not known, and should be the focus of future studies.

Growing evidence suggesting that CLA promotes notable changes in the gut microbial composition have led to the hypothesis that the targeted perturbation of the gut microbiome could increase endogenous CLA production in the gut, which could in turn provide metabolic benefits to the host. In one such study, C57Bl/6J mice were given a daily oral gavage with the 10,12 CLA-producing bacterial strain *Lactobacillus plantarum* PL62 (10^7^ and 10^9^ colony forming units (CFU)), while consuming a high fat diet for eight weeks. Mice given either the low or the high dose of PL62 gained less weight on the high-fat diet with an equivalent food intake, exhibited lower plasma triglycerides and leptin, and trended towards smaller fat pads than the control mice [228]. In the only known similar study in humans, obese women were given the prebiotic inulin-type fructans (ITF; 16 g/day of a 50:50 ratio of inulin:oligofructose) for three months [239]. While the ITF consumption did not promote any measurable weight loss, it significantly increased the prevalence of CLA-producing bacteria in the gut, including species of *Bifidobacteria* and *Lactobacillus* [239]. Moreover, the circulating levels of 9,11 CLA and 10,12 CLA were inversely correlated with the plasma lipids and hemoglobin A1c (HbA1c) levels, respectively, suggesting that the enrichment for CLA-producing gut microbes could reduce cardiovascular and diabetes risk factors, such as hyperlipidemia and insulin resistance [239]. To date, no studies have examined what effect dietary CLA would have on endogenous CLA production by gut microbes (i.e., whether dietary CLA delivered to the gut microbiota would synergize with gut bacterial production of CLA). The notion that the endogenous production of CLA by gut microbes could improve rather than promote insulin resistance has important and novel translational implications for future efforts to treat human disease.

## 5. Conclusions

Initially identified as a fatty acid with potent anti-carcinogenic properties, research on the health effects attributed to CLA has evolved to include anti-obesogenic and anti-atherosclerotic tendencies. The literature to date suggests that CLA, and primarily the 10,12 CLA isomer, consistently confers some degree of body weight and/or adiposity loss in animal models and humans. However, such effects on body energetics may not impart protection against obesity-associated comorbidities, such as type 2 diabetes. It is also clear that CLA is protective against atherosclerosis in several animal models, but its efficacy against human cardiovascular disease is less clear. Finally, whether the effects of CLA on body weight and/or cardiovascular disease are mediated by direct changes in the gut microbial composition and/or metabolites remains to be determined. As public interest in nutraceuticals such as CLA grows, more studies are urgently needed in order to validate their efficacy and safety in pre-clinical and human trials.

## Figures and Tables

**Table 1:** Conjugated linoleic acid (CLA) studies in humans with the endpoint of body weight/composition.

Study	Study Participants	Treatment Groups	Study Design	Effects due to CLA
[118]	Healthy men and women (*n* = 76).	Mixed CLA, 5 g/day (*n* = 38). Placebo (*n* = 38).	Randomized, double-blind, crossover, 14 weeks during moderate resistance training.	↑ muscle mass ↓ fat mass and % ↑ strength
[119]	Healthy overweight men and women (*n* = 134).	Mixed CLA-TG (*n* = 44) or CLA-FFA (*n* = 44), 3.4 g/day; placebo (*n* = 37).	Randomized, double-blind, 12–24 months	↓ serum cholesterol ↑ serum Lp(a) ↓serum leptin ↓body weight, BMI, fat ↓food intake
[115]	Healthy overweight men and women (*n* = 180).	Mixed CLA-TG (*n* = 60) or CLA-FFA (*n* = 61), 3.4 g/day; placebo (*n* = 59).	Randomized, double-blind, 12 months	↓body weight, BMI, fat
[120]	Healthy men and women (*n* = 55).	Mixed CLA, 3.2 g/day (*n* = 20), 6.4 g/day (*n* = 18); placebo (*n* = 17).	Randomized, double-blind, 12 weeks	↑ lean body mass ↑ serum CRP, IL-6
[121]	Lean (*n* = 13) and obese (*n* = 12) young men (23–38 years). Lean (*n* = 20) and obese (*n* = 14) older men, 50–64 years).	Mixed CLA (3 g/day) + PUFA (3 g/day); placebo (3 g palm/soy oil/day).	Randomized, double-blind, crossover, 12 weeks with a 12-week washout.	↓ fat mass in young/obese group ↑ lean mass in young/obese group ↑ adiponectin in young groups
[113]	Healthy overweight men and women (*n* = 52).	Mixed CLA (1.7, 3.4, 5.1, or 6.8 g/day); placebo (9 g olive oil/day).	Randomized, double-blind for 12 weeks	↓ fat mass (<3.4 g/day) ↑ lean mass (<3.4 g/day)
[122]	Healthy men and women of stable weight (*n* = 80).	Mixed CLA (1.7 g/day) or placebo.	Randomized, double-blind for 12 weeks	↓ body weight, BMI, body fat, waist-to-hip ratio, subcutaneous fat
[123]	Postmenopausal women with T2DM (*n* = 35).	Mixed CLA (8 g/day), or placebo (safflower oil).	Randomized, double-blind, crossover, 16 weeks with a 4-week washout.	↓ body weight, BMI, trunk adiposity
[124]	Healthy weight-stable men and women (*n* = 64) who regularly exercise.	Mixed CLA (3.9 g/day), or placebo (sunflower oil).	Randomized, double-blind, 12 weeks	No effects on body weight ↓ plasma cholesterol and insulin (women only)
[125]	Overweight hyperlipidemic males (*n* = 28).	Mixed CLA (3.5 g/day), 9,11 CLA (3/5 g/day), or placebo (safflower oil).	Randomized, double-blind, crossover, 8 weeks. with a 4-week washout.	No effects on body weight ↓ plasma TNF
[116]	Healthy, overweight men and women (*n* = 40).	Mixed CLA (4 g/day) or placebo (safflower oil).	Randomized, double-blind, 6 months.	↓ body weight, BMI, fat mass ↑ resting metabolic rate and activity level
[126]	6–10-year-old children (boys and girls) with a BMI >85^th^ percentile (*n* = 53).	Mixed CLA (3 g/day) or placebo.	Randomized, double-blind, 6 months.	↓ body fat, BMI ↑ lean body mass ↓ HDL cholesterol
[127]	Obese women (*n* = 28).	Mixed CLA (3.2 g/day) or placebo (olive oil).	Randomized, double-blind, 8 weeks with concurrent exercise.	None
[128]	Moderately overweight, borderline hyperlipidemic men and women (*n* = 15).	Mixed CLA (1.3 g/day), 9,11 CLA (1.3 g/day), or placebo.	Randomized, double-blind, crossover, 8-week treatment period with a 4-week washout.	No effects on body weight or plasma lipids.
[129]	Moderately obese weight-stable men and women (*n* = 122).	Mixed CLA (3.4 g/day), or placebo (olive oil).	Randomized, double-blind. Subjects consumed a low-calorie diet for 8 weeks to achieve weight loss, then took CLA or placebo for 52 weeks.	No effects on body weight re-gain with CLA.
[117]	Men and women with signs of metabolic syndrome (*n* = 60).	Mixed CLA (3 g/day) or placebo.	Randomized, double-blind, 12 weeks.	↓ body fat
[130]	Obese men (*n* = 25).	9,11 CLA (3 g/day), or placebo (olive oil).	Randomized, double-blind, 12 weeks.	↑ insulin resistance ↑ fat mass
[131]	Overweight but weight stable men and women (*n* = 19).	Mixed CLA (4 g/day) or placebo (safflower oil).	Randomized, double-blind, 6 months.	↓ body weight ↑ fat oxidation (during sleep)
[132]	Postmenopausal women (*n* = 81).	Mixed CLA (5.5 g/day), 9,11 CLA (4.7 g/day), or placebo (olive oil).	Randomized, double-blind, 16 weeks.	↓ total body fat and lower body fat.
[133]	Abdominally obese older men (*n* = 25).	Mixed CLA (4.2 g/day), or placebo.	Randomized, double-blind, 4 weeks.	↓ sagittal abdominal diameter
[134]	Men with signs of metabolic syndrome (*n* = 60).	10,12 CLA (3.4 g/day), or placebo.	Randomized, double-blind, 12 weeks.	↑ insulin resistance and glycemia ↓ HDL cholesterol
[135]	Overweight men and women (*n* = 54).	Mixed CLA (either 1.8 or 3.6 g/day), or placebo (oleic acid).	Randomized, double-blind for 13 weeks. Subjects consumed a very-low-calorie diet for 3 weeks leading up to study.	No effect of CLA on body weight regain vs. placebo. ↓ appetite ↑ satiety
[136]	Overweight and obese men (*n* = 16).	CLA-enriched butter (2% total fat), control butter (0.1% CLA). Test butters were ~24% total calories consumed.	Randomized, double-blind, crossover, 4-week treatment period with a 8-week washout.	No difference in abdominal adipose tissue.
[137]	Healthy men and women (*n* = 40).	Mixed CLA (4.5 g/day), or placebo (olive oil).	Randomized, double blind, 12 weeks.	No effect on body weight, BMI, or body fat. ↓ limb skin fold thickness ↓ brachial artery flow-mediated dilation.
[138]	Women with metabolic syndrome (*n* = 14).	Microencapsulated mixed CLA (3 g/day), or placebo.	Randomized, 90 days, in conjunction with a low-calorie diet.	↓ plasma insulin levels. ↓ body fat mass No effect on plasma lipids.
[139]	Sedentary males (*n* = 18).	Mixed CLA (3 g/day) or placebo.	Randomized, double-blind, 4 weeks, concurrently with exercise.	No difference in body weight, fat, or BMI. ↓ plasma triglycerides, VLDL, LDL, leptin, and insulin.
[140]	Overweight and obese women (*n* = 74).	Mixed CLA (3 g/day) or placebo (sunflower oil).	Randomized, double-blind, 12 weeks.	↓ hip circumference. No effect on body weight, BMI, or waist circumference.
[141]	Obese children, ages 8–18 (*n* = 50).	Mixed CLA (3 g/day), metformin (1 g/day), or placebo.	Randomized, double-blind, 16 weeks.	↑ insulin sensitivity.

CLA: Conjugated linoleic acid; BMI: body mass index; T2DM: type 2 diabetes mellitus; CLA-TG: conjugated linoleic acid triglyceride; CLA-FFA: conjugated linoleic acid free fatty acid; HDL: high-density lipoproteins; VLDL: very low-density lipoproteins; CRP: C-reactive protein; IL: interleukin; LDL: low-density lipoproteins; TNF: tumor nucrosis factor; ↑: increased; ↓: decreased.

**Table 2:** Human studies with endpoints related to atherosclerosis or cardiovascular disease.

Study	Study Participants	Treatment Groups	Study Design	Effects due to CLA
[202]	Overweight and obese men and women (*n* = 401).	80% 9,11 CLA + 20% 10,12 CLA (4 g/day), or placebo.	Randomized, double-blind, 6 months.	No effect on body weight. No effect on aortic stiffness, blood pressure, plasma lipids, CRP, or HOMA-IR.
[113]	Healthy overweight men and women (*n* = 52).	Mixed CLA (1.7, 3.4, 5.1, or 6.8 g/day); placebo (9 g olive oil/day).	Randomized, double-blind for 12 weeks.	↓ HDL cholesterol
[203]	Overweight men (*n* = 85).	Mixed CLA (4.5 g/day), or placebo (safflower oil).	Randomized, double-blind for 4 weeks.	↓ body weight. ↓ blood pressure. No impairment of endothelial function.
[204]	Healthy men (*n* = 49).	9,11 CLA (3 doses: 0.6, 1.2, and 2.4 g/day) or 10,12 CLA (0.6, 1.2, or 2.4 g/day).	Randomized, double-blind, crossover for 8 weeks for each isomer dose consecutively (6 months per isomer), with a 6-week washout between isomers.	No effect on body weight or composition. ↑ LDL:HDL and total cholesterol:HDL (10,12 CLA).
[205]	Healthy men (*n* = 32).	9,11 CLA (1.421 g/day) or placebo (milk).	Randomized, double-blind, crossover for 6 weeks, with a 7-week washout.	No effect on blood lipids.
[206]	Healthy young men (*n* = 38).	Mixed CLA (5.5 g/day), or placebo.	Randomized, double-blind, for 5 weeks.	No effect on body weight or blood lipids. ↑ urine lipid peroxidation products.
[137]	Healthy men and women (*n* = 40).	Mixed CLA (4.5 g/day), or placebo (olive oil).	Randomized, double blind, 12 weeks.	No effect on body weight, BMI, or body fat. ↓ limb skin fold thickness ↓ brachial artery flow-mediated dilation.
[207]	Healthy women (*n* = 74).	Mixed CLA (3 g/day) or placebo.	Randomized, double-blind, 12 weeks.	No effect on CRP or ADMA levels.
[208]	Normolipidemic men and women (*n* = 57).	Mixed CLA (3 g/day), 80% 9,11 CLA + 20% 10,12 CLA (3 g/day), or placebo (linoleic acid).	Randomized, double-blind, 8 weeks.	↓ plasma triglycerides and VLDL. No effect on body weight, plasma glucose, or insulin.
[209]	Healthy young men (*n* = 60).	Mixed CLA (5.5 g/day), control diet.	Randomized, double-blind, 5 weeks.	No effects on blood pressure or arterial elasticity.
[210]	Healthy middle-aged men and women (*n* = 92) with LDL phenotype B.	9,11 CLA (3 g/day), 10,12 CLA (3 g/day), or placebo.	Randomized, double-blind, 13 weeks.	No changes in plasma LDL, HDL, triglycerides, glucose, or insulin.
[211]	Healthy middle-aged men (*n* = 30).	Mixed CLA (2.2 g/day) or placebo.	Randomized, double-blind, 8 weeks.	No effects on plasma inflammatory markers.
[212]	Healthy non-diabetic men and women (*n* = 45).	80% 9,11 CLA + 20% 10,12 CLA (4 g/day) or placebo.	Randomized, double-blind, crossover for 2 weeks, with a 4-week washout.	No effects on platelet function.

CLA: conjugated linoleic acid; LDL: low-density lipoprotein; HDL: high density lipoproteins; CRP: C-reactive protein; VLDL: very low-density lipoproteins; ADMA: asymmetrical dimethylarginine; BMI: body mass index; HOMA-IR: homeostatic model assessment of insulin resistance; ↑: increased; ↓: decreased.

## Abbreviations

Conjugated linoleic acid (CLA); cis-9, trans-11 CLA (9,11 CLA); trans-10, cis-12 CLA (10,12 CLA); Generally Regarded As Safe (GRAS); Polyunsaturated fatty acids (PUFA); uncoupling protein 1 (UCP1); peroxisome proliferator activated receptor gamma (PPARγ); body mass index (BMI); metabolic syndrome (MetS); C-reactive protein (CRP); homeostatic model assessment of insulin resistance (HOMA-IR); low-density lipoprotein receptor-deficient mice (*Ldlr*^−/−^); apoE-deficient mice (*apoE*^−/−^); very low-density lipoproteins (VLDL); low-density lipoproteins (LDL); high density lipoproteins (HDL); ATP binding cassette transporter A1 (ABCA1); apolipoprotein A1 (ApoA1); cluster of differentiation 36 (CD36); vascular cell adhesion molecule-1 (VCAM-1); intercellular adhesion molecule-1 (ICAM-1); very-late antigen 4 (VLA-4); macrophage-1 antigen (Mac-1); cluster of differentiation 18 (CD18); C-X-C chemokine receptor 4 (CXCR4); classically-activated macrophage (M1); alternatively-activated macrophage (M2); metabolically-activated macrophage (MMe); arginase-1 (ARG-1); early growth response protein-2 (EGR-2); peripheral arterial tonometry (PAT); platelet-activating factor acetylhydrolase (PAF-AH); asymmetrical dimethylarginine (ADMA); high sensitivity C-reactive protein (hs-CRP); short chain fatty acids (SCFA); inulin-type fructans (ITF); hemoglobin A1c (HbA1c).
